# Dried small fish provide nutrient densities important for the first 1000 days

**DOI:** 10.1111/mcn.13192

**Published:** 2021-05-04

**Authors:** Kendra A. Byrd, Lauren Pincus, Monica M. Pasqualino, Farayi Muzofa, Steven M. Cole

**Affiliations:** ^1^ WorldFish Bayan Lepas Malaysia; ^2^ Center for Human Nutrition, Department of International Health Johns Hopkins Bloomberg School of Public Health Baltimore Maryland USA; ^3^ WorldFish Lusaka Zambia; ^4^ International Institute of Tropical Agriculture Dar es Salaam Tanzania

**Keywords:** complementary feeding, dietary strategies, essential fatty acids, lactation, low‐income countries, micronutrients, pregnancy

## Abstract

Inadequate nutrient intakes are prevalent among many populations in sub‐Saharan Africa and increasing fish consumption among pregnant/lactating women and children is one strategy to improve diets and address nutrient deficiencies. We report the nutrient content of two fish‐based recipes—fish powder and fish chutney—that contain dried small fish available in local markets in Zambia. The contribution of a serving of each recipe to the recommended daily intakes of iron, zinc, calcium and docosahexaenoic acid (DHA) for pregnant/lactating women and children 6–24 months was calculated because these nutrients are commonly deficient in African diets. We found that one 10‐g serving of fish powder provides 20% or more of the daily calcium recommendation and 37% or more of the daily DHA recommendation for both pregnant/lactating women and children. A 30‐g serving of fish chutney provides over 40% of the daily calcium recommendation for pregnant women and over 50% for lactating women. Additionally, we investigated the nutrient density (nutrients per kilocalorie) of the fish powder and compared it with the nutrient density of a small‐quantity lipid‐based nutrient supplement plus (SQ‐LNS‐plus). SQ‐LNS‐plus is designed to enhance children's diets by providing micronutrients and DHA. Fish powder is similar to SQ‐LNS‐plus in iron and zinc density and even higher in calcium and DHA density. Consuming dried small fish as part of a daily meal can be a viable strategy for combatting nutrient deficiencies in the first 1000 days.

**Key messages**

- Fish‐based recipes that use locally available dried small fish have the potential to address nutrient deficiencies that often occur in the first 1000 days of life.
- Fish powder is a concentrated source of iron, zinc, calcium and omega‐3 fatty acids, which can greatly improve the quality of a meal when added in small amounts.
- Leveraging locally available and culturally acceptable foods, such as small fish, is an important consideration when designing programmes to address malnutrition in low‐ and middle‐income countries.

## 1 INTRODUCTION

Consuming animal‐source foods (ASF) during the critical window of pregnancy, lactation and up to 2 years of life (also known as the first 1000 days) is a key strategy for breaking the cycle of malnutrition (Iannotti, 2018; Karakochuk et al., 2017; Lee et al., 2013). Increasing the consumption of ASF among vulnerable populations in low‐ and middle‐income countries is critical to improving dietary quality. Children in Zambia, for instance, often consume below the recommended amounts of calcium, iron and vitamin B12 (Caswell et al., 2018), and the diets of rural women are often lacking in almost all micronutrients (Kaliwile et al., 2019). Inadequate dietary intake contributes to stunting in children, which is associated with increased morbidity and mortality (Victora et al., 2008), and afflicts over 40% of children under 5 years old in Zambia (Global Nutrition Report—Zambia Nutrition Profile, 2020). Given the high poverty levels in Zambia (Chibuye, 2014; Mason et al., 2020), women, infants and children need greater access to nutrient‐dense ASF to break the cycle of malnutrition.

Pregnant and lactating women have slightly higher energy needs, and substantially higher nutrient needs than other women do, in order to support the development of a healthy fetus and to compensate for the increased demand of milk production (Karakochuk et al., 2017). Nutrient deficiencies and inadequate gestational weight gain during pregnancy are associated with poor perinatal outcomes (Adu‐Afarwuah et al., 2017), thus pregnant and lactating women benefit from consuming foods that add both kilocalories (kcals) and nutrients to their prepregnancy diets. Fish chutney, a condiment that can be added to women's regular meals (rather than a replacement for any food on the plate), is a potential avenue for increasing energy (kcals) and nutrient intakes during pregnancy and lactation. In Bangladesh, research on locally made fish chutney designed for pregnant and lactating women found that both the nutrient and kcal content was appropriate to address their increased needs (Bogard et al., 2015). Thus, it is plausible that recipes using an available and acceptable fish could play the same role in Zambia.

Fish, an important ASF for resource‐poor populations, is consumed and accepted as part of the local diet in Zambia (Longley et al., 2014), and small fish from freshwater fisheries are often the most affordable of the ASF (Funge‐Smith & Bennett, 2019). However, the frequency of fish intake varies among infants and young children. Children living closer to fisheries consume fish more frequently than children who live further from fisheries (O'Meara et al., 2021), indicating an opportunity to increase fish intake among certain populations of children. Small fish from capture fisheries in and outside of Zambia are sometimes underfished (Kolding et al., 2019) and are thus a promising option for sustainably increasing the supply of fish. Furthermore, small fish consumed whole are more nutrient dense than the fish fillet (i.e., the muscle). Although the fillet has several micronutrients and fatty acids, fish that are prepared and consumed whole, or with only the intestine removed, can provide a greater quantity of iron (Roos et al., 2007) and calcium (Isaacs, 2016) per serving. Small fish are also often an important source of omega‐3 fatty acids. For example, *usipa* (*Engraulicypris sardeila*), a small pelagic fish found in Lake Malawi, is high in the omega‐3 fatty acid docosahexaenoic acid (DHA) (Byrd et al., 2020). Increasing the consumption of small fish by women and children in the first 1000 days is plausible from a supply and affordability standpoint and has the potential to increase nutrient intakes.

Fish powder in particular is a product that could increase the nutrient intakes of infants and young children because moisture is removed during the drying process and nutrients are concentrated. The nutrient density (nutrient per kcal) of the foods consumed by infants 6–11 months old is especially important given their high nutrient needs relative to their low caloric needs (Dewey, 2013; Dewey, 2016). This is true even in breastfed children, because the amount of iron and zinc in breast milk is relatively low (Dewey, 2013). Thus, specialized complementary food products are designed to provide an optimal ratio of nutrients to Kcals for infants and children (Arimond et al., 2015). Specialized complementary food products are supplements that contain different formulations (often using peanuts as a base) are and designed to be added to infants and children's meals at home. Given what is known about the nutrient composition of small fish, fish powder using these species may have a nutrient density that is comparable to specialized complementary food products, while also having the additional benefits of being locally available and culturally acceptable (Longley et al., 2014).

Our study compares the nutrient density of a locally produced fish powder to SQ‐LNS‐plus, a specialized complementary food product, to add to the information on the range of tools low‐ and middle‐income countries can deploy to address malnutrition. Although there is a clear role for specialized complementary food products, foods that utilize small fish, such as fish powders, have not been adequately investigated as a potential alternative.

The aim of this study was to assess the nutrient content of two locally developed fish‐based recipes—fish powder and fish chutney—and to calculate the contribution of calcium, zinc, iron and DHA to the diets of pregnant and lactating women and children 6–24 months. We also compared the nutrient density of iron, zinc, calcium and DHA of fish powder with SQ‐LNS‐plus. Our findings will contribute evidence to guide the formulation of policies and practices intended to improve the diets of resource‐poor women and children.

## 2 MATERIALS AND METHODS

We chose iron, zinc and calcium as micronutrients of interest given they are often found to be inadequate in the diets of women and children in low‐ and middle‐income countries (Beal et al., 2017; Ferguson et al., 2016). Among the essential fatty acids, we chose DHA because fish is often a good source and DHA concentrations have been found to be deficient in blood samples among certain populations in South Africa and Tanzania (Stark et al., 2016).

### 2.1 Development of fish‐based recipes

The fish powder and fish chutney recipes developed for this study were part of a larger development project to address food and nutrition security in the Mbala and Luwingu Districts of Zambia's Northern Province (Mulungu et al., 2017). These two recipes were selected from the project as they were relatively simple and cost effective to prepare, and utilized a small list of locally available ingredients.

**FIGURE 1:**
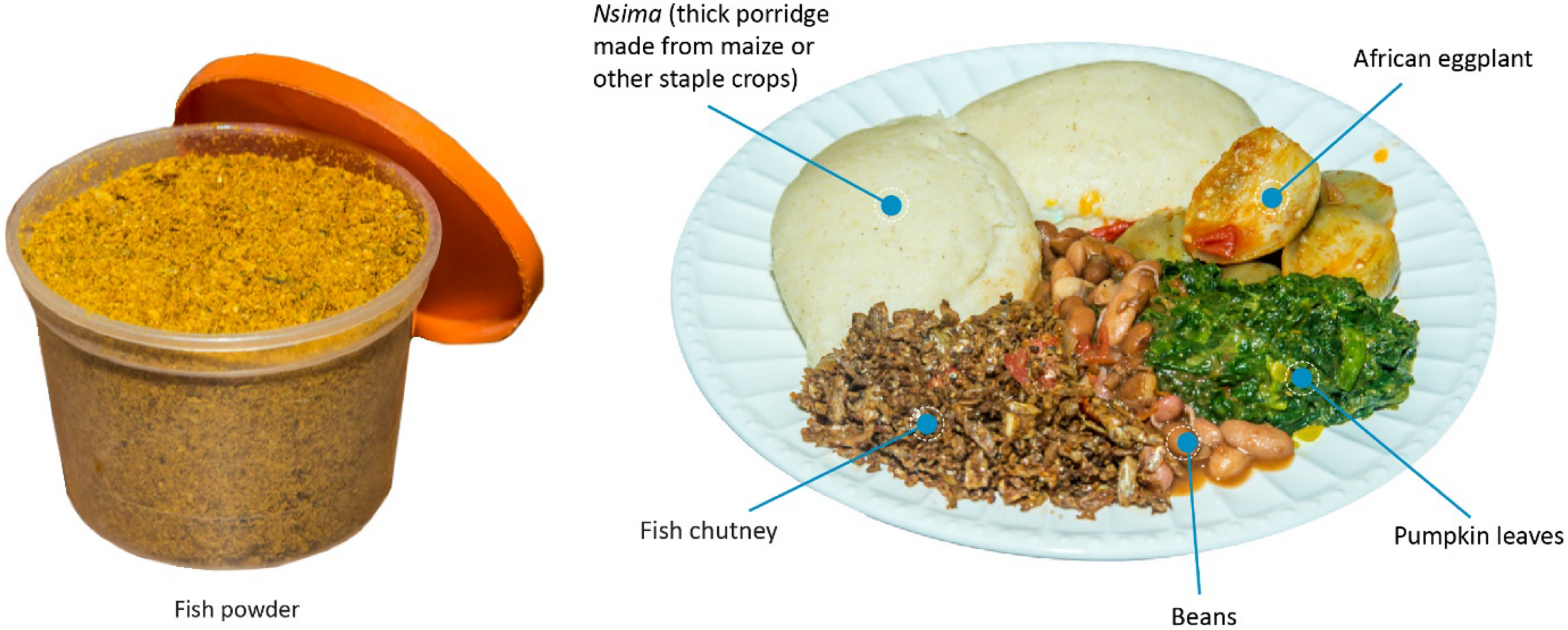
Fish powder pictured alone (left) and fish chutney (right) pictured as part of a Zambian meal

Small fish were chosen for the recipes due to their high nutrient content and because they are generally available, affordable and acceptable in Zambia (Longley et al., 2014). *Dagaa* (*Rastrineobola argentea*) was selected for the fish powder, and *inkundu* (*Pseudocronilabrus philander*) was selected for the fish chutney, given their availability in the districts where the recipes were developed. It was assumed that if the *inkundu* and *dagaa* fish‐based foods were acceptable as ingredients, then other small fish would also be acceptable, as there are many small indigenous species found in this region. Known as *dagaa* in Zambia and Tanzania, *omena* in Kenya and *mukene* in Uganda, this freshwater small pelagic fish species is endemic to Lake Victoria (Isaacs, 2016). *Inkundu* is another freshwater small species of cichlid found in the southern Africa region (Fish Base, 2020). Both small fish species are commonly consumed dried and whole in the Zambian context (Kolding et al., 2019; Nölle et al., 2020). The other ingredients in the fish powder and fish chutney were selected to increase the flavour and acceptability of the recipes (Table 1). See Figure 1 for a picture of the fish powder and the fish chutney, with the fish chutney pictured as an accompaniment to a Zambian meal.

**TABLE 1:** Ingredients and preparation methods of the fish‐based recipes

	Fish powder (serving size: 10 g)	Fish chutney (serving size: 30 g)
Ingredients	2 tablespoons roasted dried small fish powder 4 teaspoons salt 1 teaspoon turmeric 1/2 teaspoon chilli powder	10 tablespoons dried small fish 4 tablespoons pounded raw groundnuts 10 tablespoons chopped onion 1 teaspoon chilli powder 2 teaspoons salt 4 teaspoons vegetable oil
Preparation	• Rinse the dried small fish • Remove the fish from the water, roast for 30 min over low heat • Remove from heat, let cool • Pound with a mortar and pestle, and sieve the resulting fish powder until smooth • In a small bowl, thoroughly mix the dried fish powder, salt, turmeric powder and chilli powder • Store in an airtight container	• Rinse the dried small fish • Remove the fish from the water, roast for 30 min over low heat and then coarsely pound • In a pan, heat oil then add coarsely pounded fish and fry over medium heat for 15 min • Add onions and continue frying for another 10 min • Add pounded groundnuts and cook for 15 min or until mixture stops sticking • Add salt and chilli powder and then fry for 5 min • Remove from heat and leave to cool • Store in an airtight container

The fish‐based recipes received high scores for taste and acceptability using organoleptic evaluations (WorldFish, 2017). The recipes were tested with women and men in Mbala and Luwingu, two districts in the Northern Province of Zambia. Approximately 40% of the population of Northern Province lives in the lowest wealth quintile, and 56% of women and 64% of men are employed in agriculture (Zambia Statistics Agency, 2020). The recipes were cooked according to the instructions and provided as an accompaniment to vegetable dishes. For this study, we considered both recipes appropriate for consumption by pregnant and lactating women. However, we did not consider the chutney recipe appropriate for infants given the instructions to ‘coarsely pound’ the dried fish, which may result in bones remaining in the chutney. Therefore, we did not include it in any recommended daily intakecalculations for infants and children 6–24 months.

We analysed the serving sizes that were included in the WorldFish recipe booklet which was 10 g for the fish powder and 30 g for the fish chutney (WorldFish, 2017).

### 2.2 Analysis of nutrient composition

A batch of each fish‐based recipe was prepared in a standard Zambian kitchen using only locally available cooking implements (mortar and pestle, stove and spatula) to ensure the recipes could be produced at the household level. The fish were purchased dried from local markets. One 100‐g sample of each recipe was prepared. The nutrient composition of the two fish‐based recipes was analysed at the Microchem Specialized Lab Services in Cape Town, South Africa, in 2017. Recipes were homogenized prior to sampling. Only one sample was tested per fish recipe due to resource constraints. The amount of energy was determined using chemical analysis (Harold et al., 1981). Protein, carbohydrates, fats and minerals were quantified using standard methods per the Association of Official Analytical Chemists (AOAC) (Horwitz et al., 1970). Protein was measured using AOAC method 968.06, carbohydrates using 982.14, fats using 996.06 and minerals using 984.27. The minerals were measured using the inductively coupled plasma emission spectroscopic method. Omega‐3 and omega‐6 fatty acids were quantified using gas chromatography.

### 2.3 Methods for calculating the fulfilment of the fish‐based recipes to estimated RNIs

We assessed the nutrient content of the recipes relative to the recommended nutrient intakes (RNIs) for iron and zinc and the recommended intakes for calcium and DHA from the WHO/FAO guidelines for vitamins and minerals (WHO & FAO, 2004) and fatty acids (FAO & WHO, 2010), which provide the latest understanding of the estimated requirements of nutrients for healthy individuals. We calculated the estimated per cent of the recommended intakes fulfilled by 10 g of fish powder for pregnant and lactating women, infants 6–11 months and children 12–24 months. For the fish chutney, we calculated the estimated per cent of the recommended intakes fulfilled by 30 g of the recipe for pregnant and lactating women only.

No specific RNI for iron in pregnant women is listed in the FAO/WHO guidelines; therefore, we used the value of 29.4 mg/day given for women aged 18–50. This value could be considered a corollary to the Recommended Dietary Allowance (RDA) of 27 mg/day for pregnant women based on the Institute of Medicine guidelines (Institute of Medicine [US] Panel on Micronutrients, 2001). For the iron recommendation for all age groups, we chose the values based on 10% bioavailability to account for both the anti‐nutrient (i.e., phytate) composition of the Zambian diet (Caswell et al., 2018) and the positive effect of fish on iron bioavailability (Michaelsen, Hoppe, Roos, Kaestel, Stougaard, Lauritzen, et al., 2009).

For zinc, we used the RNI values for pregnant and lactating women, infants and young children as published (WHO & FAO, 2004). The zinc values for pregnant women are divided by trimester. The first year of lactation is also divided up into three distinct time periods. We chose the values based on low bioavailability of zinc, in accordance with other dietary analyses conducted in Zambia (Caswell et al., 2018; Gibson et al., 2018). For calcium, we used the mean estimated recommended intakes as published (WHO & FAO, 2004).

Pregnant and lactating women should consume 200 mg/day of DHA according to the most recent FAO/WHO guidelines (FAO & WHO, 2010). For infants and children 6–23 months, the recommendation is an intake of 10‐ to 12‐mg/kg body weight per day (FAO & WHO, 2010). Based on a previous study (Bogard et al., 2017), we used a figure of 110‐mg DHA per day for infants and young children, representing the midpoint of the recommended range of intakes based on body weights at the 50th percentile of children between 7 and 23 months (WHO, 2006).

### 2.4 Selection of a specialized complementary food product

We selected a small‐quantity lipid‐based nutrient supplement plus (SQ‐LNS‐plus) produced by DSM Nutritional Products Ltd, a company headquartered in The Netherlands (Smuts et al., 2019), to compare with the fish powder recipe. The SQ‐LNS‐plus product we selected for comparison in our study included micronutrients found in previous versions of LNS, with the addition of DHA (indicated by the ‘plus’ in the product name) to promote cognitive development (Smuts et al., 2019). SQ‐LNS‐plus, in addition to other LNS products, have been shown to be effective in increasing iron status and reducing anaemia (Smuts et al., 2019; Stewart et al., 2019). To our knowledge, SQ‐LNS‐plus is not currently distributed in Zambia. However, we selected this specialized complementary food product because a product with an enhanced DHA density is a relevant comparison to a fish powder containing naturally occurring DHA.

We then made the comparison of the fish powder to both established iron, zinc and calcium nutrient densities desired for complementary foods and to the nutrient densities of the SQ‐LNS‐plus. Desired nutrient densities have been delineated based on infant stomach capacity, kilocalorie needs and the need to avoid displacing breast milk (Dewey & Brown, 2003).

## 3 RESULTS

### 3.1 Nutrient composition and densities of the fish‐based recipes

**FIGURE 2:**
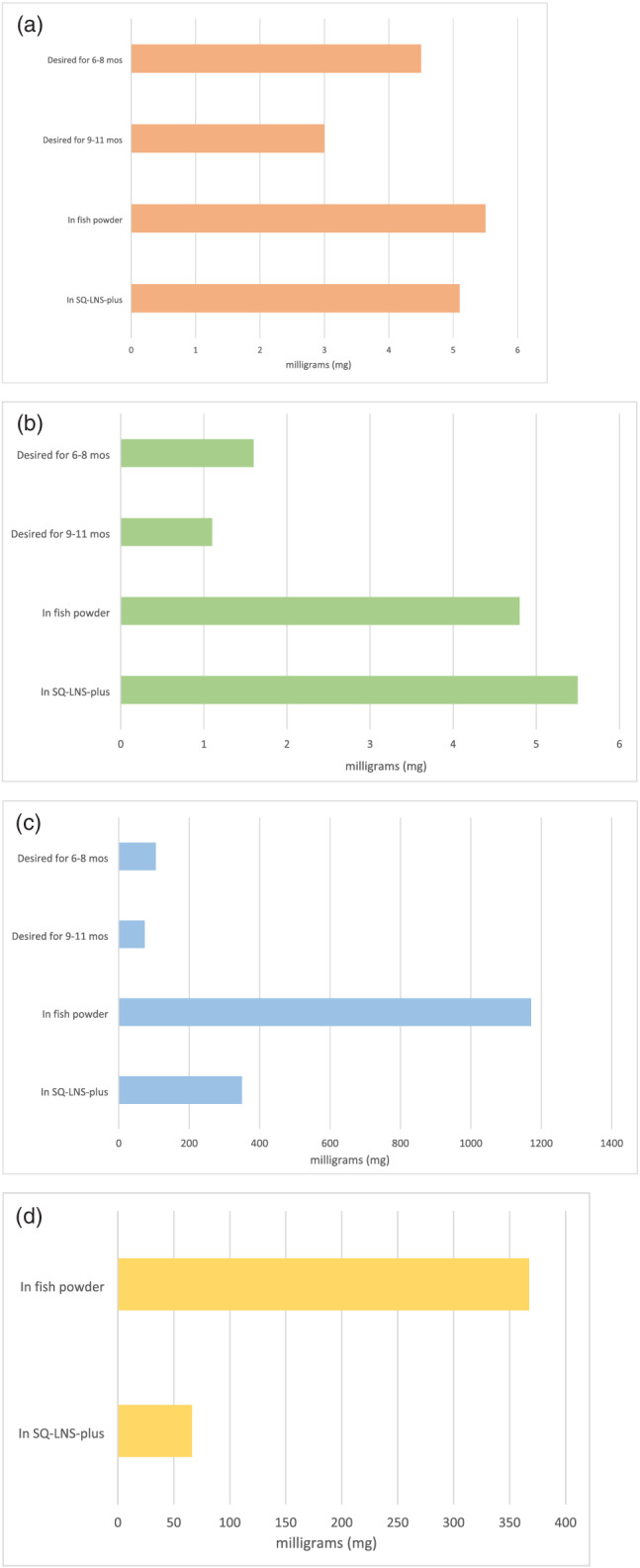
(a) The amount of iron in milligrams (mg) desired per 100 kcal in complementary foods for both the 6–8 months (mos) period and the 9–11 months period in infants (Dewey & Brown, 2003). The amount of iron per 100 kcal in fish powder made from *dagaa* and in small‐quantity lipid‐based nutrient supplement plus (SQ‐LNS‐plus) is also shown. Exact values can be found in Table S1. (b) The amount of zinc in milligrams (mg) desired per 100 kcal in complementary foods for both the 6–8 months (mos) period and the 9–11 months period in infants (Dewey & Brown, 2003). The amount of zinc per 100 kcal in fish powder made from *dagaa* and in SQ‐LNS‐plus is also shown. Exact values can be found in Table S1. (c) The amount of calcium in milligrams (mg) desired per 100 kcal in complementary foods for both the 6–8 months (mos) period and the 9–11 months period in infants (Dewey & Brown, 2003). The amount of calcium per 100 kcal in fish powder made from *dagaa* and in SQ‐LNS‐plus is also shown. Exact values can be found in Table S1. (d) A comparison of milligrams (mg) of docosahexaenoic acid (DHA) per 100 kcal of fish powder and in SQ‐LNS‐plus. Exact values for (a)–(d) can be found in Table S1

The nutrient composition for both the fish powder and fish chutney by serving size can be seen in Table 2. A 10‐g serving of fish powder provides 20 kcal, 1.1 mg of iron, 1.0 mg of zinc and 234 mg of calcium. These values achieve the respective desired nutrient densities for complementary food for infants 6–11 months (Dewey & Brown, 2003) (Figure 2a–c). The fish powder and the SQ‐LNS‐plus both exceed the nutrient densities recommended for iron, zinc and calcium, but the fish powder is approximately three times denser than the SQ‐LNS‐plus for calcium.

**TABLE 2:** Nutrient content of fish‐based recipes per recommended serving size

Nutrient	Fish powder (10 g prepared)	Fish chutney (30 g prepared)	Recommended intakes for infants 6–12 months, per day	Recommended intakes for children 1–3 years, per day	Recommended intakes for pregnant women, per day	Recommended intakes for lactating women, per day
Energy (kcal)	19.9	99.8	Recommended intakes fluctuate based on energy intakes from breast milk.^a^		Recommended intakes vary based on physical activity levels.	
Fat (g)	0.51	1.93	30^e^	No data		
Protein (g)	3.58	10.8	11	13	71	71
Carbohydrates (g)	0.04	7.95	95	130	175	210
Omega‐3 fatty acids (mg)	122	213	Values dependent on energy intakes			
ALA	13.4	213	Values dependent on energy intakes			
EPA	0.00	0.00	No data	No data	100	100
DHA	73.5	0.00	110	110	200	200
Omega‐6 fatty acids (mg)	50.0	510	Values dependent on energy intakes			
Minerals (mg)						
Calcium	234	510	400	500	1200	1000^b^
Iron	1.10	1.91	9.3	5.8	29.4	15
Zinc	0.96	2.20	4.1	4.1	5.5, 7.0, 10.0^c^	9.5, 8.8, 7.2^d^
Sodium	1066	312	370^e^	800^e^	1500^e^	1500^e^

*Note*: Recommended intake values for protein and carbohydrates for infants, children and women and recommended energy intake for women were obtained from the Dietary Reference Intake (DRI) report (Food and Nutrition Board, Institute of Medicine, and National Academies, 2005). Recommended intake values for total fat, omega‐3 and omega‐6 fatty acids were obtained from the FAO/WHO report on fatty acids (FAO & WHO, 2010), with exception for recommended total fat intake for 6–11‐month‐olds, which was obtained from the DRI report. For infants and young children, see table 6.1. For adults, see table 5.1. Recommended intake values for calcium, iron and zinc were obtained from FAO/WHO reports. Values for iron and zinc assumed 10% bioavailability and moderate bioavailability, respectively (WHO & FAO, 2004).

Abbreviations: ALA, alpha‐linolenic acid; EPA, eicosapentaenoic acid; DHA, docosahexaenoic acid.

^a^ For precise values, see Dewey and Brown (2003).

^b^ Value only available for the third trimester of pregnancy (WHO & FAO, 2004).

^c^ Values reported for the first, second and third trimesters, respectively, assuming moderate bioavailability (WHO & FAO, 2004).

^d^ Values reported for 0–3, 3–6, and 7–12 months of lactation, respectively, assuming moderate bioavailability (WHO & FAO, 2004).

^e^ Values are adequate intakes (AI) taken from DRI report (Food and Nutrition Board, Institute of Medicine, & National Academies, 2019). An AI value means that there was not enough evidence to make a more precise recommendation.

The fat in the fish powder is primarily essential fatty acids; one serving contains 50 mg of omega‐6 fatty acids and 121 mg of omega‐3 fatty acids (Table 2). The most predominant omega‐3 fatty acid in the fish powder is DHA at 74 mg, and although the SQ‐LNS‐plus was enriched with fish oil, the fish powder has a higher density of DHA (368‐mg DHA/100 kcal) than the SQ‐LNS‐plus (66‐mg DHA/100 kcal) (Figure 2d). The sodium content of 10 g of fish powder is 1064 mg, which is approximately three times the amount that children under 1 year old need at that age (Food and Nutrition Board, Institute of Medicine, & National Academies, 2019).

The fish chutney contains 1.9 mg of iron, 2.2 mg of zinc and 510 mg of calcium per 30‐g serving (Table 2). One 30‐g serving has nearly 100 kcal, with protein (at 11 g) providing the bulk of the kilocalories. The chutney also provides nearly 2 g of fat. Of the essential fatty acids, a serving of fish chutney provides mostly omega‐6 fatty acids; however, there are 213 mg of omega‐3 fatty acids in the form of alpha‐linolenic acid (ALA). There are 0 g of DHA in this recipe, likely due to the fact that the fish chutney used *inkundu* whereas the fish powder used *dagaa*, and the content of DHA varies in dried small fish (Nölle et al., 2020).

### 3.2 The role of the fish‐based recipes in fulfilling the RNI among pregnant and lactating women

**FIGURE 3:**
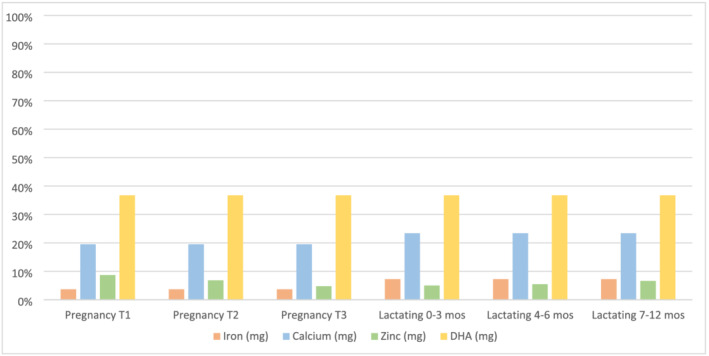
Estimated contribution (%) to recommended daily intakes of iron, zinc, calcium and docosahexaenoic acid (DHA) from a 10‐g serving of fish powder. The pregnancy and lactation periods are broken down by the three trimesters of pregnancy (T1, T2 and T3) and by month of lactation, given that zinc recommendations vary between these time periods. The recommended intakes for iron, zinc and calcium are from the most recent WHO/FAO guidelines for vitamins and mineral intakes (WHO & FAO, 2004) and for fatty acid intakes (FAO & WHO, 2010). For the iron bioavailability calculations, we used 10%, and low bioavailability was used for the zinc calculations, based on current consumption patterns in Zambia

We found that even small servings of the fish‐based recipes add substantial nutrients to the diets of pregnant and lactating women. The recipes provide additional iron, zinc, calcium and DHA—key nutrients for the growth and development of the fetus (Karakochuk et al., 2017). One serving (10 g) of fish powder fulfilled 4% and 7% of the daily iron recommendation for pregnant and lactating women, respectively (Figure 3). For the recommended daily zinc intake, the fish powder fulfilled approximately 9% of the needs of pregnant women in the first trimester, which decreased to 5% in the third trimester as zinc needs increase. During early lactation, a serving of fish powder fulfilled 5% of the recommendation, which increased slightly given that zinc needs decline as the lactation period goes on. A 10‐g serving of fish powder met one fifth or more of the calcium recommendation for pregnant and lactating women at 20% and 23%, respectively. A 10‐g serving of fish powder provided 37% of the estimated DHA requirement for both pregnant and lactating women.

**FIGURE 4:**
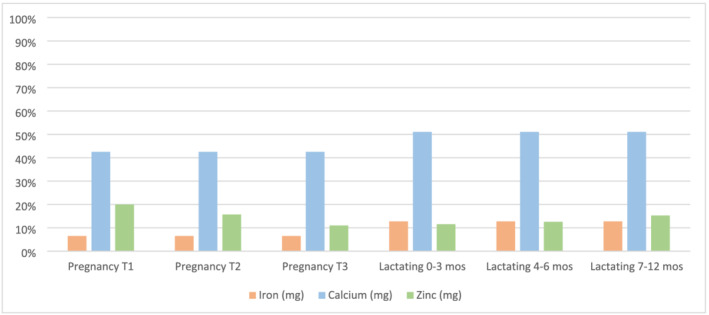
Estimated contribution (%) to recommended daily intakes of iron, zinc and calcium from a 30‐g serving of fish chutney made from *inkundu*. The pregnancy and lactation periods are broken down by the three trimesters of pregnancy (T1, T2 and T3) and by month of lactation, given that zinc recommendations vary between these time periods. The recommended intakes for iron, zinc and calcium are from the most recent WHO/FAO guidelines for vitamins and mineral intakes (WHO & FAO, 2004). For the iron bioavailability calculations, we used 10%, and low bioavailability was used for the zinc calculations, based on current consumption patterns in Zambia

The fish chutney, designed to be consumed as a condiment, provided 7% of the iron recommendation for pregnant women and 13% for lactating women (Figure 4). It provided slightly more zinc than the fish powder, with one serving fulfilling 20% of the zinc recommendation for pregnant women in their first trimester and decreasing to 11% in the third trimester. During lactation, the fish chutney fulfilled between 12% and 15% of the zinc recommendation. Similar to the fish powder, a small amount of fish chutney fulfilled a large portion of calcium recommendation for both pregnant and lactating women. One serving provided 43% of the calcium recommendation for pregnant women and 51% for lactating women. However, the fish chutney, when made using the fish (*inkundu*) listed in the recipe booklet, contained no DHA.

### 3.3 The role of the fish‐based recipes in fulfilling the RNI among infants and young children

**FIGURE 5:**
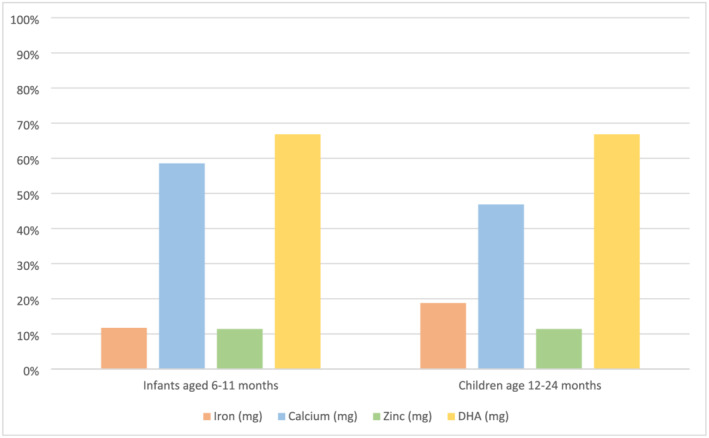
Estimated contribution (%) to infant and children recommended daily intakes of iron, zinc, calcium and docosahexaenoic acid (DHA) from a 10‐g serving of fish powder. The recommended intakes for iron, zinc and calcium are from the most recent WHO/FAO guidelines for vitamins and mineral intakes (WHO & FAO, 2004) and for fatty acid intakes (FAO & WHO, 2010). For the iron bioavailability calculations, we used 10%, and low bioavailability was used for the zinc calculations, based on current consumption patterns in Zambia

We also found that a small amount of fish powder can improve the diets of infants and young children by providing additional iron, zinc, calcium and DHA to a given meal. A single 10‐g serving of fish powder provided 12% of the iron recommendation for infants 6–11 months and 19% for children 12–24 months (Figure 5). Put another way, the addition of a spoonful of fish powder to three meals a day could fulfil over one third of the iron recommendation for a 6 to 11 month old and 60% of the iron recommendation for a 1 year old, providing a boost of iron to the diet for relatively few kilocalories. For infants and children 6–24 months, adding a 10‐g portion of fish powder to a meal would fulfil an estimated 11% of their zinc recommendation, without a high risk of displacing kilocalories from breast milk or other complementary foods. If consumed with three meals a day, fish powder would provide a third of the daily zinc recommendation for infants and children, even when children are consuming a diet resulting in a low bioavailability of zinc (i.e., a maize‐based diet). One serving of fish powder provided nearly 60% of the daily calcium recommendation for infants under 12 months and 47% for children 12–24 months. The fish powder also provided 67% of the DHA recommendation for 6‐ to 24‐month‐olds. If consumed only twice in a day, the fish powder would completely fulfil the DHA recommendation for infants and young children.

## 4 DISCUSSION

Our study found that fish‐based recipes have a high potential to address inadequate nutrient intakes in Zambia. Processing the dried small fish into powder achieved the optimum nutrient density of iron, zinc and calcium for the diets of children between 6 and 11 months old, a stage when adequate nutrient intake is highly critical and difficult to achieve. We also found that dried small fish in the form of fish powder fulfils a large percentage of women and children's daily calcium and DHA nutrient recommendation. Fish chutney can provide additional calcium, and some iron and zinc, to the diets of pregnant and lactating women, even when consumed in small amounts as a condiment. In places where dried small fish are readily available, national nutrition programmes can promote the consumption of this natural resource as a means of reducing nutrient deficiencies among nutritionally vulnerable populations.

Nutrient deficiencies in infants and young children can lead to lifelong growth impairments, thus obtaining enough nutrients from the diet is paramount during the first 1000 days of life (Karakochuk et al., 2017). Calcium is needed in many cellular processes and is imperative for the development of healthy bones and teeth (WHO & FAO, 2004). Deficiencies in zinc result in an impaired immune response and suboptimal growth, whereas deficiencies in iron can impair cognitive development and, if chronic, can lead to anaemia (Grantham‐McGregor & Ani, 2001; WHO & FAO, 2004). Calcium, iron and zinc are often limited in the diets of children in sub‐Saharan Africa, and increasing children's consumption of small fish has been recommended to fill the gaps (Caswell et al., 2018; Ferguson et al., 2019). Previous studies have documented that adding dried fish to foods is a viable method for increasing key nutrients in the diet (Bogard et al., 2015; Folake & Otegbayo, 2006; Omueti et al., 2009). However, most of these studies did not report the nutrient densities of fish‐based products. Our findings demonstrate that even when dried small fish are included in relatively easy‐to‐prepare recipes, they can increase the amount of calcium, iron and zinc in children's diets, likely without displacing breast milk.

In addition to micronutrients, the fish powder used in this recipe provides a good source of essential fatty acids. The density of DHA in the fish powder was higher than the density of DHA in SQ‐LNS‐plus, which had been fortified with fish oil (Smuts et al., 2019). This result strengthens the evidence that small fish can be an excellent source of DHA (Byrd et al., 2020). Because the fish for the recipe was purchased dried and pounded into powder in a home, we show that much of the DHA is preserved through drying and pounding. Thus, the fish powder recipe presented here has the potential to increase DHA in the diet of both women and children. Maternal intakes of DHA have been associated with improved cognition in children (Hibbeln et al., 2019).

Despite the high amounts of nutrients found in the fish powder, the nutrients absorbed by the body are reliant on several factors. There is potential for anti‐nutrients, such as phytates or polyphenols found in plant‐based foods, to block the absorption of iron or zinc (Gibson et al., 2018), depending on with what the fish powder is served during mealtime. However, fish has an ‘enhancing factor’ for iron, which leads to increased absorption (Michaelsen, Hoppe, Roos, Kaestel, Stougaard, Lauritzen, et al., 2009). Additionally, the calcium consumed in whole small fish is inherently high (due to the preservation of bones in the fish) and is absorbed at a similar rate as the calcium in dairy products (Isaacs, 2016). Furthermore, complementary food products with a peanut base, such as SQ‐LNS, contain phytates that affect iron and zinc absorption (Michaelsen, Hoppe, Roos, Kaestel, Stougaard, Lauritzen, Mølgaard, et al., 2009), whereas fish powder contains little to no phytates. Thus, although the fish‐based recipes only fulfil a small part of the RNI for iron and zinc, their percentages could be increased by either increasing the serving size or increasing the potential for absorption by consuming the recipes with roots, tubers or leafy vegetables, rather than maize‐based foods.

Although the fish powder provides a high density of iron, zinc, calcium and DHA, the high sodium content is potentially of concern in this recipe, and therefore, the salt can be omitted if desired or salted to taste before or after serving. This modification will not impact the iron, zinc, calcium or DHA content, though it may impact acceptability. The chili powder can also be omitted without impacting the nutrient content if the chilli powder proves to be too spicy for children's palates. However, the turmeric should be retained as it does provide iron to the fish powder at a concentration of 0.36 mg per quarter teaspoon (USDA, 2017). The serving size can also be modified. Although we analysed the serving sizes as published in the Zambia fish‐based recipes booklet (WorldFish, 2017), the serving size of either the fish powder or fish chutney can be modified according to taste, nutritional needs or economic practicalities. Programmes, or individual households, may consider increasing the serving size of fish powder for women, for example, to increase their nutrient intakes beyond the 10‐g dose. One benefit of using a locally available food to promote a healthy diet is that the recipes and serving sizes can be modified at home to suit the needs and capabilities of individual households.

Randomized controlled trials investigating the impact of fish and fish products on children's nutrient status are few and provide mixed results. Whereas some studies have shown no effect of fish‐based products on children's nutrient status (Lartey et al., 1999; Lin et al., 2008), other studies have shown an improvement in children's calcium and vitamin D status in Nigeria (Thacher et al., 2015) and zinc status in Indonesia (Ikawati et al., 2020). It remains an open question whether integrating a nutrient‐dense food such as fish powder into children's diets will improve their nutrient status.

Fish consumption in children is associated with a reduction in stunting, as demonstrated by a cross‐sectional study in Zambia of urban children under 5 years old (Marinda et al., 2018) and by an analysis of fish consumption and stunting in 49 low‐ and middle‐income countries (Headey et al., 2018). However, evidence from impact evaluations is mixed. Recent findings from Cambodia demonstrated that small fish can replace milk powder in products designed for treating malnutrition, given they are both micronutrient‐dense foods (Sigh et al., 2018). Another study in Cambodia found that fish are largely on par with milk in complementary food products for promoting growth (Skau et al., 2013). Other trials in Kenya and Ghana did not see an impact on child growth when fish were added to complementary foods (Konyole et al., 2019; Lartey et al., 1999). Further evidence in the Zambian setting on the role of fish powder on nutrition outcomes in infants and children is warranted, and closer attention should be paid to the food matrix through which the fish is delivered, given many complementary foods contain phytates.

Our analysis showed that the fish chutney has the potential to boost women's calcium intake but does not fulfil much of pregnant and lactating women's RNI for iron and zinc. Fish chutney also provides 100 kcal per serving and thus can be promoted alongside other iron‐ and zinc‐rich foods commonly consumed in resource‐poor areas in Zambia. Additionally, considering calcium plays a critical role in preventing preeclampsia among pregnant women (Khaing et al., 2017), a recipe that increases calcium intake should not be overlooked.

Although specialized complementary food products such as SQ‐LNS likely have a role to play in alleviating nutrient deficiencies, strategies leveraging locally available nutrient‐dense foods are needed as well. In this study, we provide evidence that fish‐based recipes have the potential to address nutrient deficiencies. Specialized complementary food products and recipes made with locally available foods both have strategic advantages and disadvantages. The former are susceptible to non‐adoption by rural or urban resource‐poor populations, given they are new food products that these populations have not seen or consumed before (Daly et al., 2015). However, specialized complementary foods are often prepared to be ready‐to‐eat, thus reducing women's or other caregivers' time burdens. The fish‐based recipes in our study utilize relatively few ingredients that are available in local Zambian markets and were found to be acceptable by rural consumers (WorldFish, 2017); however, the ingredients still need to be bought and recipes prepared by rural households. The evidence presented here provides a model through which locally available, nutrient‐dense foods can be leveraged to improve diets in the first 1000 days, while recognizing that fish‐based recipes can take on a number of formulations and should be adapted to the local context. Context‐specific, participatory research would provide insights on additional ways to utilize fish in diets that may be more efficient.

In addition to the issue of the time burden to prepare the recipes is the issue of cost. Freshwater small fish are often the most affordable of the ASF (Funge‐Smith & Bennett, 2019), and families can also purchase small fish in various amounts, as budgets allow, as opposed to a larger purchase of a big fish (Belton & Thilsted, 2014; Isaacs, 2016). Thus, a homemade product that can be added to other complementary foods is likely to be less expensive than LNS, which would need to be sold at an unsubsidized price of 1.05 USD, at least in Ethiopia (Segre et al., 2015). According to the recipe booklet, 12 servings of fish chutney cost the equivalent of 0.40 USD to make and a reported 0.13 USD to make four servings of fish powder (WorldFish, 2017). This results in a price of about 0.03 USD per serving for both recipes. Though prices vary, the relative low cost of the fish‐based recipes is a promising feature.

Our study is not without limitations. Due to budget constraints, we were not able to analyse a full panel of nutrients or collect information on microbial contamination in the recipes. Future studies might analyse the concentration of vitamin B12 in fish powder and chutney, because indigenous small fish have been shown to be an important source (Thilsted et al., 2016). Additionally, the nutrient content measured in these two recipes included small fish widely available and consumed in the northern part of Zambia, yet these are only two species out of many that are harvested and consumed. Thus, fish powder made with fish other than *dagaa*, or fish chutney made with fish other than *inkundu*, will have varying nutrient contents. Thus, it would have been useful to analyse both recipes made with both types of fish, but the recipes were developed based on what was available in the market in two separate locations at the time—*dagaa* in the Mbala district and *inkundu* in the Luwingu district. There is evidence that marine fish species with similar characteristics (e.g., fish length) have comparable nutrient compositions (Hicks et al., 2019), though this same analysis is not yet available for freshwater fish.

## 5 CONCLUSION

Diets in many parts of Zambia provide low amounts of nutrients that are critical for early growth and development. Incorporating nutrient‐dense ASF, such as dried small fish, into recipes made with locally available ingredients is a promising strategy to alleviate nutrient deficiencies. Processing dried small fish into powder form is a viable way to increase the densities of nutrients in a given meal. Fish powder can provide an equal, or greater, density of iron, zinc, calcium and DHA than some specialized complementary food products and can be produced at home. Given the promising nutrient density of fish‐based recipes, it would be advantageous for both policy makers and civil society to promote recipes such as those presented in this study as a way to complement other approaches used to improve malnutrition within the first 1000 days of life. Additionally, programmes that work with households to promote the nutritional benefits of consuming small fish and preparing fish‐based recipes, such as those tested in this study, can help resource‐poor consumers take advantage of food resources in new ways.

## CONFLICTS OF INTEREST

The authors declare no conflict of interest.

## CONTRIBUTIONS

KAB, LP, SMC and MMP conceptualized the study. SMC and FM developed the methodology. KAB and LP performed the formal analysis. KAB, LP, SMC and MMP wrote and prepared the original draft. KAB, LP, SMC, FM and MMP wrote, reviewed and edited the manuscript. KAB performed the visualization. KAB supervised the study. SMC did the funding acquisition. All authors have read and agreed to the published version of the manuscript.

## Supporting information

**Table S1:** Calcium, iron, and zinc requirements per 100 kilocalories used for calculations in Figures 2a‐d

## Data Availability

The data that support the findings of this study are available from the corresponding author upon reasonable request.
