# Supplementary material for: Dried small fish provide nutrient densities important for the first 1000 days
Source: Matern Child Nutr. 2021 May 4;17(4):e13192. doi: 10.1111/mcn.13192 (PMC8476445; doi:10.1111/mcn.13192)
Supplement: Supplementary file 1 — Table S1: Calcium, iron, and zinc requirements per 100 kilocalories used for calculations in Figures 2a‐d [file MCN-17-e13192-s001.docx]

**Supplementary Materials for manuscript titled: “**Dried small fish provide nutrient densities important for the first 1,000 days”

Table S1: Calcium, iron, and zinc requirements per 100 kilocalories used for calculations in figures 2a-d

| Per 100 kilocalories | Iron (mg) | Zinc (mg) | Calcium (mg) | DHA^1^ (mg) |
| --- | --- | --- | --- | --- |
| Desired amount for 6-8 month-old infants^2^ | 4.5 | 1.6 | 105 | Not specified |
| Desired amount for 9-11 month-old infants | 3.0 | 1.1 | 74 | Not specified |
| In fish powder | 5.5 | 4.8 | 1171 | 367.5 |
| In SQ-LNS-plus^3^ | 5.1 | 5.5 | 350 | 66.3 |

^1^Docosahexaenoic acid

^2^Values taken from Dewey and Brown, 2003

^3^Small-quantity lipid-based nutrient supplement plus
